# Obstetric Clinic

**Published:** 1902-03

**Authors:** 


Obstetric Clinic. By Denslow Lewis, Ph. C., M. D., Professor of Gynecology in the Chicago Polyclinic; President of the Attending Staff of Cook County Hospital, Chicago; President of the Chicago Medical Examiners' Association; Vice President of the Illinois State Medical Society; Ex-President of the Physicians' Club of Chicago; Consulting Obstetrician to the Florence Nightingale Home; Senior Gynecologist and Obstetrician to the Lakeside Hospital, Chicago; late Special Commissioner from the Illinois State Board of Health and the Health Department of Chicago for the Investigation of Municipal Sanitation in European Cities. Illustrated. 640 octave pages. Price, $3.00 net. Published by E. H. Colegrove, 65 Randolph St., Chicago.

This book consists of thirty-nine clinical lectures on practical obstetrics, delivered to students and practitioners in the Cook County Hospital, Chicago. It includes also a discussion of criminal abortion, infanticide, illegitimacy, the restriction of venereal diseases, the regulation of prostitution, and other medico-sociologic topics. The author's experience and long service as a teacher in this line, make his work an authority that will be appreciated by the profession. T. J. B.

